
JOURNAL INFORMATION
==============================
NLM Title Abbreviation: ScientificWorldJournal
Journal ID: ScientificWorldJournal
NLM Journal ID: 101131163
Journal ID: TSWJ

The Scientific World Journal

ISSN: 2356-6140
EISSN: 1537-744X
Publisher: Wiley

ARTICLE INFORMATION
==============================
PMCID: PMC5901334
PMID: 18167611
DOI: 10.1100/tsw.2007.313
Article ID: 932030
Article version: 1
Subjects: Directions in Science

Heparanase, A Key Target for Gene Therapy against Human Malignancies

Zhang Youlei 1
Wang Yi 1
Fu Zhiren 2
Shen Feng 1 *
1 Eastern Hepatobiliary Surgery Hospital, Second Military Medical University, Shanghai, 200438, China
2 Liver Transplantation Center of Changzheng Hospital, Second Military Medical University, Shanghai, 200003, China
*Corresponding author.Academic Editor: Martin Goette

Publication date: 2007
Electronic publication date: 2007 Dec 17
Volume: 7
First page: 1965
Last page: 1967
Received 2007 Oct 4; Revised 2007 Nov 28; Accepted 2007 Dec 4
Copyright: Copyright © 2007 Youlei Zhang et al.
Copyright year: 2007
License: This is an open access article distributed under the Creative Commons Attribution License, which permits unrestricted use, distribution, and reproduction in any medium, provided the original work is properly cited.
License URL: https://creativecommons.org/licenses/by/3.0/

Keywords: heparanase, antisense oligodeoxynucleotide, small interfering RNA, angiogenesis, invasion, metastasis, human malignancies, cancer therapy

==============================
Directions in Science
*TheScientificWorldJOURNAL* (2007) 7, 1965–1967
ISSN 1537-744X; DOI 10.1100/tsw.2007.313

TheScientificWorldJOURNAL
www.thescientificworld.com

### Heparanase, A Key Target for Gene Therapy against Human Malignancies

Youlei Zhang[1], Yi Wang[1], Zhiren Fu[2], and Feng Shen[1,*]

*[1]Eastern Hepatobiliary Surgery Hospital, Second Military Medical University, Shanghai, 200438, China; [2]Liver Transplantation Center of Changzheng Hospital, Second Military Medical University, Shanghai, 200003, China*

**E-mail:** zhangylei111@hotmail.com

*Received October 4, 2007; Revised November 28, 2007; Accepted December 4, 2007; Published December 17, 2007*

**KEYWORDS**: heparanase, antisense oligodeoxynucleotide, small interfering RNA, angiogenesis, invasion, metastasis, human malignancies, cancer therapy

Invasion and metastasis are key features of many human malignant tumors. In the process of invasion and metastasis, tumor cells must break through the barrier of the extracellular matrix (ECM) and the basement membrane (BM). The key in this procedure is the ECM-degrading enzymes secreted by tumor cells. The major component of ECM and BM is heparan sulfate proteoglycan (HSPG), which is mainly composed of a protein core and several heparan sulfate (HS) side-chains[1]. Heparanase, an endoglycosidase, is a novel ECM-degrading enzyme that cleaves HS at several sites. It induces the degradation of the ECM and BM, facilitates the migration of tumor cells, and releases HS-bound active fibroblast growth factor (FGF) and vascular endothelial growth factor (VEGF), and, therefore, promotes tumor invasion, metastasis, and angiogenesis[2,3]. Heparanase is aberrantly expressed by many human malignancies, such as gastrointestinal carcinoma, hepatocellular carcinoma, breast carcinoma, metastatic melanoma, and carcinomas of ovary, prostate, bladder, and pancreas[4,5,6,7,8,9,10,11]. In addition, the heparanase expression level is closely correlated to the invasive and metastatic potency of the tumor cells and the prognosis of the cancer patients[7,8,9,10,11]. By virtue of its important roles in the process of tumor progression, heparanase has been considered a key target for antitumor drug development[1,2,3].

It has been reported that nonanticoagulant species of heparin, several sulfated oligosaccharides, and sulfated polysaccharides can inhibit the enzymatic activity of heparanase and, therefore, inhibit tumor invasion, metastasis, and angiogenesis[12,13,14,15]. One representative of these inhibitors, a sulfated oligosaccharide named PI-88, is being tested in multicenter clinical trials[14]. However, recent studies indicate that heparanase can also promote tumor progression independently by its nonenzymatic activity through activating phosphatidylinositol 3-kinase (PI3K)/protein kinase B (PKB/AKT) and p38 signaling pathways, inducing the expression of cyclooxygenase 2 (COX-2), VEGF, and FGF2, or promoting adhesion of tumor cells to ECM[16,17,18,19,20]. These nonenzymatic functions of heparanase may contribute to the limited efficiency of heparanase enzymatic inhibitors in cancer therapy.

Due to the potential nonspecific and enzymatically limited inhibitory activities of these inhibitors, as well as the great difficulties in identifying efficient inhibitors, genetic approaches targeting heparanase have been regarded as promising alternatives[21,22,23,24,25]. Uno et al. constructed an adenoviral vector carrying an antisense, full-length, human heparanase cDNA and found that the heparanase expression, the *in vitro* invasiveness, and *in vivo* metastasis of human lung cancer A549 cells were specifically

*Corresponding author.
©2007 with author.
Published by TheScientificWorld; www.thescientificworld.com inhibited[22]. A similar study also found that the inhibition of heparanase expression was efficacious in the prevention and treatment of melanoma metastasis[23]. A recent study by Zhang et al. shows that genetic inhibition of human heparanase by specific antisense oligodeoxynucleotide (AS-ODN) or small interfering RNA (siRNA) can efficiently regress the invasion, metastasis, angiogenesis, and *in vivo* tumor growth of human hepatocellular carcinoma[24]. Together with another report demonstrating that AS-ODN against human heparanase inhibits the expression of heparanase and the *in vitro* invasiveness of human mammary carcinoma cells in a dose-dependent manner[25], accumulating evidence have suggested that small inhibitory genetic molecules (e.g., AS-ODN and siRNA) targeting heparanase are of great value in the control and treatment of human malignancies.

Although the genetic blocking molecules have been shown to inhibit the expression of heparanase and regress tumor progression, the methods of stable transfection of these molecules have some limits for clinical application, such as gene integration, expression efficiency, and drug withdrawal difficulties. Alternatively, the transient transfection of some small genetic molecules, such as AS-ODN and siRNA, is more similar to the regular drug administration. For example, they can be easily synthesized, administered, and withdrawn[26,27,28]. AS-ODN is a potential therapeutic agent that can inhibit gene expression in a sequence-specific manner[26,27]. RNA interference (RNAi) is a post-transcriptional mechanism of gene silencing through chromatin remodeling, inhibition of protein translation, or direct mRNA degradation[28]. Data from the recent studies have demonstrated that down-regulation of the expression of heparanase by either AS-ODN or siRNA can efficiently inhibit the invasion and metastasis, as well as angiogenesis of human hepatocellular carcinoma and mammary carcinoma[24,25]. Therefore, AS-ODN and siRNA specific for human heparanase may be of potential value in the treatment of human cancers.

The genetic blocking methods employing AS-ODN and siRNA are powerful technologies that hold great promise in cancer therapy. Nevertheless, further investigations are required to overcome the obstacles for their successful clinical application, such as the delivery efficiency, nonspecific immune responses, incomplete gene suppression, and even the off-target effects.

### ACKNOWLEDGMENTS

We thank Dr. Chen T. for critical reading and kind help in editing of the manuscript.

### REFERENCES

1. Sasisekharan, R., Shriver, Z., Venkataraman, G., and Narayanasami, U. (2002) Roles of heparan-sulphate glycosaminoglycans in cancer. *Nat. Rev. Cancer* **2**, 521–528.
2. Parish, C.R., Freeman, C., and Hulett, M.D. (2001) Heparanase: a key enzyme involved in cell invasion. *Biochim. Biophys. Acta* **1471**, M99–M108.
3. Vlodavsky, I., Goldshmidt, O., Zcharia, E., Atzmon, R., Rangini-Guatta, Z., Elkin, M., Peretz, T., and Friedmann, Y. (2002) Mammalian heparanase: involvement in cancer metastasis, angiogenesis and normal development. *Semin. Cancer Biol.* **12,** 121–129.
4. Vlodavsky, I., Friedmann, Y., Elkin, M., Aingorn, H., Atzmon, R., Ishai-Michaeli, R., Bitan, M., Pappo, O., Peretz, T., Michal, I., Spector, L., and Pecker, I. (1999) Mammalian heparanase: gene cloning, expression and function in tumor progression and metastasis. *Nat. Med.* **5,** 793–802.
5. Hulett, M.D., Freeman, C., Hamdorf, B.J., Baker, R.T., Harris, M.J., and Parish, C.R. (1999) Cloning of mammalian heparanase, an important enzyme in tumor invasion and metastasis. *Nat. Med.* **5,** 803–809.
6. Friedmann, Y., Vlodavsky, I., Aingorn, H., Aviv, A., Peretz, T., Pecker, I., and Pappo, O. (2000) Expression of heparanase in normal, dysplastic, and neoplastic human colonic mucosa and stroma. Evidence for its role in colonic tumorigenesis. *Am. J. Pathol.* **157,** 1167–1175.
7. Zcharia, E., Metzger, S., Chajek-Shaul, T., Friedmann, Y., Pappo, O., Aviv, A., Elkin, M., Pecker, I., Peretz, T., and Vlodavsky, I. (2001) Molecular properties and involvement of heparanase in cancer progression and mammary gland morphogenesis. *J. Mammary Gland Biol. Neoplasia* **6,** 311–322.
8. Koliopanos, A., Friess, H., Kleeff, J., Shi, X., Liao, Q., Pecker, I., Vlodavsky, I., Zimmermann, A., and Buchler,

M.W. (2001) Heparanase expression in primary and metastatic pancreatic cancer. *Cancer Res.* **61**, 4655–4659.

9. Cohen, I., Pappo, O., Elkin, M., San, T., Bar-Shavit, R., Hazan, R., Peretz, T., Vlodavsky, I., and Abramovitch, R. (2006) Heparanase promotes growth, angiogenesis and survival of primary breast tumors. *Int. J. Cancer* **118**, 1609–1617.
10. EL-Assal, O.N., Yamanoi, A., Ono, T., Kohno, H., and Nagasue, N. (2001) The clinicopathological significance of heparanase and basic fibroblast growth factor expressions in hepatocellular carcinoma. *Clin. Cancer Res.* **7**, 1299–1305.
11. Bar-Sela, G., Kaplan-Cohen, V., Ilan, N., Vlodavsky, I., and Ben-Izhak, O. (2006) Heparanase expression in nasopharyngeal carcinoma inversely correlates with patient survival. *Histopathology* **49**, 188–193.
12. Parish, C.R., Coombe, D.R., Jakobsen, K.B., Bennett, F.A., and Underwood, P.A. (1987) Evidence that sulphated polysaccharides inhibit tumor metastasis by blocking tumor-cell-derived heparanase. *Int. J. Cancer* **40**, 511–558.
13. Vlodavsky, I., Mohsen, M., Lider, O., Svahn, C.M., Ekre, H.P., Vigoda, M., Ishai-Michaeli, R., and Peretz, T. (1994) Inhibition of tumor metastasis by heparanase inhibiting species of heparin. *Invasion Metastasis* **14,** 290–302.
14. Parish, C.R., Freeman, C., Brown, K.J., Francis, D.J., and Cowden, W.B. (1999) Identification of sulfated oligosaccharide-based inhibitors of tumor growth and metastasis using novel in vitro assays for angiogenesis and heparanase activity. *Cancer Res.* **59**, 3433–3441.
15. Miao, H.Q., Elkin, M., Aingorn, E., Ishai-Michaeli, R., Stein, C.A., and Vlodavsky, I. (1999) Inhibition of heparanase activity and tumor metastasis by laminarin sulfate and synthetic phosphorothioate oligodeoxynucleotides. *Int. J. Cancer* **83**, 424–431.
16. Imada, T., Matsuoka, J., Nobuhisa, T., Okawa, T., Murata, T., Tabuchi, Y., Shirakawa, Y., Ohara, N., Gunduz, M., Nagatsuka, H., Umeoka, T., Yamamoto, Y., Nakajima, M., Tanaka, N., and Naomoto, Y. (2006) COX-2 induction by heparanase in the progression of breast cancer. *Int. J. Mol. Med.* **17**, 221–228.
17. Zetser, A., Bashenko, Y., Edovitsky, E., Levy-Adam, F., Vlodavsky, I., and Ilan, N. (2006) Heparanase induces vascular endothelial growth factor expression: correlation with p38 phosphorylation levels and Src activation. *Cancer Res.* **66**, 1455–1463.
18. Gingis-Velitski, S., Zetser, A., Flugelman, M.Y., Vlodavsky, I., and Ilan, N. (2004) Heparanase induces endothelial cell migration via protein kinase B/Akt activation. *J. Biol. Chem.* **279**, 23536–23541.
19. Goldsmith, O., Zcharia, E., Abramovitch, R., Metzger, S., Aingorn, H., Friedmann, Y., Schirrmacher, V., Mitrani, E., and Vlodavsky, I. (2002) Cell surface expression and secretion of heparanase markedly promote tumor angiogenesis and metastasis. *Proc. Natl. Acad. Sci. U. S. A.* **99**, 10031–10036.
20. Zcharia, E., Zilka, R., Yaar, A., Yacoby-Zeevi, O., Zetser, A., Metzger, S., Sarid, R., Naggi, A., Casu, B., Ilan, N., Vlodavsky, I., and Abramovitch, R. (2005) Heparanase accelerates wound angiogenesis and wound healing in mouse and rat models. *FASEB J.* **19**, 211–221.
21. Edovitsky, E., Elkin, M., Zcharia, E., Peretz, T., and Vlodavsky, I. (2004) Heparanase gene silencing, tumor invasiveness, angiogenesis, and metastasis. *J. Natl. Cancer Inst.* **96,** 1219–1230.
22. Uno, F., Fujiwara, T., Takata, Y., Ohtani, S., Katsuda, K., Takaoka, M., Ohkawa, T., Naomoto, Y., Nakajima, M., and Tanaka, N. (2001) Antisense-mediated suppression of human heparanase gene expression inhibits pleural dissemination of human cancer cells. *Cancer Res.* **61,** 7855–7860.
23. Roy, M., Reiland, J., Murry, B.P., Chouljenko, V., Kousoulas, K.G., and Marchetti, D. (2005) Antisense-mediated suppression of heparanase gene inhibits melanoma cell invasion. *Neoplasia* **7**, 253–262.
24. Zhang, Y., Li, L., Wang, Y., Zhang, J., Wei, G., Sun, Y., and Shen, F. (2007) Downregulating the expression of heparanase inhibits the invasion, angiogenesis and metastasis of human hepatocellular carcinoma. *Biochem. Biophys. Res. Commun.* **358**, 124–129.
25. Zhang, Y., Fu, Z., Zhang, J., Wang, Y., and Shen, Q. (2003) Inhibition of invasiveness of human mammary carcinoma cell line MDA435 by heparanase antisense oligodeoxynucleotides. *Natl. Med. J. China* **83**, 204–207.
26. Crooke, S.T. (1999) Molecular mechanisms of action of antisense drugs. *Biochim. Biophys. Acta* **1489,** 31–44.
27. Tamm, I., Dorken, B., and Hartmann, G. (2001) Antisense therapy in oncology: new hope for an old idea? *Lancet* **358**, 489–497.
28. Pai, S.I., Lin, Y.Y., Macaes, B., Meneshian, A., Hung, C.F., and Wu, T.C. (2006) Prospects of RNA interference therapy for cancer. *Gene Ther.* **13**, 464–477.

**This article should be cited as follows:**

Zhang, Y., Wang, Y., Fu, Z., and Shen, F. (2007) Heparanase, a key target for gene therapy against human malignancies. *TheScientificWorldJOURNAL* **7**, 1965–1967. DOI 10.1100/tsw.2007.313.
